# Antigen-specific regulatory cell therapy approaches in kidney transplantation

**DOI:** 10.3389/fimmu.2026.1956545

**Published:** 2026-10-21

**Authors:** Sudipta Tripathi, Anil Chandraker

**Affiliations:** Renal Division, Department of Medicine, UMass Chan Medical School, Worcester, MA, United States

**Keywords:** alloantigen, antigen-specific cell therapy, immune regulation, immune tolerance, kidney transplantation, regulatory T cells

## Abstract

**Background:**

Antigen-specific regulatory T-cell therapies seek to control donor-reactive immunity while preserving broader immune protection. The available platforms differ substantially in cellular composition, mode of antigen recognition, manufacturing requirements, and maturity of clinical evidence.

**Scope:**

This narrative Mini Review compares donor-cell expansion, costimulation-blockade products, peptide-based targeting of indirect allorecognition, CAR- and TCR-engineered Tregs, and Tr1-enriched products. Evidence is distinguished as ex vivo or mechanistic, preclinical *in vivo*, human non-kidney, kidney feasibility and safety, or suggestive of clinical activity.

**Key insights:**

No platform is likely to suit every transplant setting. Donor-cell approaches provide broad antigenic coverage but require individualized donor material. Peptide and receptor-defined approaches offer precision and donor-independent manufacture but are constrained by antigen processing, HLA restriction, or incomplete coverage. Early CAR-Treg studies provide preliminary safety and proof-of-mechanism observations, not evidence of operational tolerance. Culture conditions, accompanying immunosuppression, and product-specific safety risks must be considered together.

**Conclusions:**

Progress will require consistent nomenclature, mechanism-linked potency assays, quantitative reporting of clinical studies, and operational measures of persistence, regulatory stability, donor-specific function, graft engagement, and clinical durability. The near-term goal is safe and sustained immunosuppression minimization. Operational tolerance must be demonstrated rather than inferred.

## 1 Introduction

Regulatory T cells (Tregs) have a central role in maintaining immune homeostasis and limiting alloimmune responses. In kidney transplantation, however, endogenous regulation is rarely sufficient to prevent rejection without pharmacologic immunosuppression. Short-term outcomes have improved substantially, but long-term graft survival remains limited by chronic alloimmune injury and by infection, malignancy, metabolic disease, and nephrotoxicity related to immunosuppressive therapy (1–4). Regulatory cell therapy therefore pursues a different goal. It seeks sustained control of donor-reactive immunity with less generalized immunosuppression.

Early kidney-transplant studies showed that autologous polyclonal Tregs and related regulatory products could be isolated, expanded, and administered without major short-term safety concerns (5–8). These small and heterogeneous studies primarily established feasibility and safety. They neither exclude a therapeutic role for polyclonal Tregs nor establish that antigen-specific products are superior. The central question is whether enrichment for donor-reactive cells increases potency, promotes localization to the allograft, permits lower cell doses, and preserves immunity to unrelated antigens. Experimental studies, including humanized models, support this possibility (9), and donor antigen-specific regulatory cells have therefore become an important area of investigation (10–12).

This review uses *regulatory cell therapy* as the broad umbrella term. *Conventional Treg product* refers to a predominantly CD4^+^FOXP3^+^ population. *Antigen-specific Treg* is reserved for a conventional Treg product with demonstrated antigen specificity. Tr1-enriched products, genetically engineered CAR- or TCR-Tregs, and heterogeneous regulatory cell lines are named separately (**Table 1**). When an original study used a product-specific term, that designation is retained and its cellular composition is stated. This framework is intended to make biological differences visible rather than impose a final nomenclature on a rapidly evolving field.

**Table 1:** Comparative characteristics and evidence maturity of regulatory cell platforms relevant to donor antigen-specific therapy in kidney transplantation.

Platform and composition	Recognition pathway/target	Donor material	HLA restriction	Genetic modification	Current evidence level	Manufacturing and translational trade-offs	Principal safety or interpretive concern
Polyclonal conventional Treg comparator Predominantly CD4^+^FOXP3^+^	No donor-specific selection. Broad endogenous TCR repertoire.	No	No	No	Kidney Phase 1/2 safety and feasibility studies (5–8). Not an antigen-specific platform.	Established isolation and anti-CD3/CD28 expansion. Larger doses may be needed because only a fraction is donor reactive.	Nonspecific suppression, contaminating effector cells, and uncertain donor-focused potency.
Donor-cell-expanded alloantigen-reactive conventional Treg	Broad intact donor antigen. Predominantly direct, with protocol-dependent indirect presentation.	Yes. Donor B cells, PBMCs, or APCs.	No predefined recipient restriction, but response reflects the donor-recipient mismatch.	No	Clinical-grade human manufacture and ex vivo evidence. Limited early clinical experience overlaps with costimulation-blockade products (17–21).	Broad coverage but individualized donor collection, variable stimulators, repeated culture, and longer manufacture.	Product heterogeneity, loss of clonotype representation, lineage instability, and contaminating effector cells.
Costimulation-blockade-derived donor-reactive product Purified or heterogeneous depending on protocol	Whole donor antigen during CD28-pathway blockade. Direct and indirect components can vary.	Usually yes	No predefined restriction	No	Kidney feasibility and safety pilot, n=3. Human liver immunosuppression-withdrawal experience, n=10 (20–22).	Shorter selection phase, but product identity is protocol dependent. Donor material and individualized manufacture remain necessary.	Effector-cell carryover, heterogeneous composition, and overinterpretation of liver evidence in kidney transplantation.
Peptide-pulsed indirect-pathway conventional Treg	Donor peptide presented by recipient HLA class II. Indirect pathway.	No viable donor cells. Donor HLA sequence is required.	Yes. Recipient HLA and selected peptide.	No	Human ex vivo and preclinical transplant-related evidence. No solid-organ infusion study identified (18, 23).	Donor-independent and precise, but peptide processing, abundance, immunodominance, and repertoire coverage must be validated.	Failure of *in vivo* target presentation, narrow coverage, and inability to encompass direct or semi-direct responses.
HLA-DR peptide-expanded ASTRL Heterogeneous regulatory cell line	Index donor HLA-DR peptide presented by recipient APCs. Indirect pathway with ex vivo linked suppression.	No viable donor cells. Donor HLA-DR sequence is required.	Yes. Recipient presentation of the selected peptide.	No	Human kidney-recipient ex vivo and multiomic studies. No clinical infusion (47, 48).	Can be manufactured after living or deceased donation without initial Treg sorting. Product includes variable CD4^+^, CD8^+^, and NK compartments.	Uncertain product identity, between-patient consistency, contribution of non-CD4 cells, dose, persistence, and clinical activity.
TCR-engineered conventional Treg	Transferred TCR recognizes donor peptide in recipient HLA. Usually indirect pathway.	No viable donor cells once target is defined.	Yes. Donor peptide and recipient HLA.	Yes	Preclinical *in vivo* transplantation evidence (24).	Defined physiological receptor sensitivity and shorter selection, but complex gene-modified manufacture and narrow eligibility.	Endogenous-transgenic TCR mispairing, unexpected specificity, vector-related risk, and target escape.
HLA-directed CAR-Treg Conventional Treg with engineered CAR	Intact donor HLA surface antigen independent of conventional TCR-MHC engagement.	No, once the donor HLA target is known.	Restricted to the selected donor-recipient mismatch.	Yes	Completed kidney Phase 1/2 study with long-term follow-up. Interim liver Phase 1/2 experience (25–27, 49–51).	Uniform high-frequency specificity and graft localization, but gene transfer, construct-specific potency assays, and long-term surveillance are required.	Unexpected antigen recognition, tonic signaling or exhaustion, immunogenicity, transgene or vector events, and incomplete donor-antigen coverage.
Tr1/T-allo10 comparator IL-10^+^FOXP3^--^enriched product	Alloantigen presented by IL-10-conditioned tolerogenic APCs.	Yes in reported T-allo10 manufacture	Donor-recipient dependent, not a single defined HLA target.	No	Human HSCT mechanistic and persistence data. No solid-organ clinical study (28).	Human evidence of alloantigen-selected clonotype persistence, but a different lineage and clinical setting.	Variable Tr1 enrichment, composition of non-Tr1 cells, and uncertain extrapolation from HSCT to kidney transplantation.

APC, antigen-presenting cell; ASTRL, antigen-specific T-cell-enriched regulatory cell line; CAR, chimeric antigen receptor; HSCT, hematopoietic stem-cell transplantation; PBMC, peripheral blood mononuclear cell; TCR, T-cell receptor; Treg, regulatory T cell; Tr1, type 1 regulatory T cell.

## 2 Literature identification and evidence classification

This narrative Mini Review was informed by targeted PubMed/MEDLINE and ClinicalTrials.gov searches updated through September 21, 2026. Search terms combined *regulatory T cell*, *antigen-specific*, *alloantigen-specific*, *kidney* or *renal transplantation*, *indirect allorecognition*, *peptide-expanded*, *costimulation blockade*, *CAR-Treg*, *TCR-Treg*, and *Tr1*. Reference lists of recent reviews and key primary studies were also examined. Trial registries, conference reports, and sponsor disclosures were used only when peer-reviewed clinical results were not available, and these sources are identified as preliminary.

Priority was given to primary human kidney-transplant studies, followed by human studies in other transplant settings and then mechanistic, ex vivo, and preclinical studies when kidney evidence was absent. Foundational studies older than 10 years were retained when necessary to establish mechanism or platform development. Throughout the review, findings are identified as mechanistic or ex vivo, preclinical *in vivo*, human non-kidney, kidney feasibility and safety, or evidence suggesting clinical activity. These categories should not be interpreted as equivalent levels of evidence.

## 3 Biological rationale for donor antigen specificity

Recipient T cells recognize alloantigen through direct, indirect, and semi-direct pathways. Direct recognition involves intact donor HLA-peptide complexes. Indirect recognition involves donor proteins processed and presented as peptides by recipient antigen-presenting cells (APCs). Semi-direct recognition occurs when recipient APCs acquire intact donor HLA-peptide complexes. The traditional distinction between an early direct response and a later indirect response is useful but incomplete. Donor extracellular vesicles can cross-dress recipient APCs, and cross-dressed dendritic cells can sustain effector responses within kidney allografts (13–15). A product directed toward one pathway may influence, but will not necessarily encompass, the full alloimmune response.

The principal theoretical advantage of antigen-specific Tregs is conditional activation. Cells that encounter their target antigen in the graft or draining lymphoid tissue can proliferate, traffic locally, and deploy suppression where it is needed. Antigen-triggered regulation may extend beyond the initiating epitope through linked suppression, in which a regulatory cell activated by one antigen suppresses responses to other antigens presented by the same APC or within the same tissue environment. Bystander regulation, competition for IL-2, CTLA-4-mediated modulation of APCs, adenosine generation, inhibitory cytokines, and recruitment of endogenous regulatory cells can further broaden the response (16). These mechanisms provide a plausible route to donor-focused regulation, but demonstration ex vivo does not establish durable clinical tolerance.

## 4 Strategies for generating antigen-specific regulatory cells

### 4.1 Donor-cell-driven expansion

Donor cell-based expansion is among the most established methods for generating antigen-specific Tregs ex vivo. An enriched Treg population is stimulated, often through repeated rounds of culture, with activated donor B cells, donor peripheral blood mononuclear cells (PBMCs), or other donor-derived APCs. Intact donor cells express multiple HLA molecules and other alloantigens. These methods therefore provide broad antigenic exposure and predominantly expand cells that recognize donor HLA through the direct pathway. Clinical-grade protocols using CD40-activated donor B cells have achieved substantial expansion while retaining suppressive activity (17, 18). Donor-reactive Tregs have also been identified in operationally tolerant kidney recipients, supporting their biological relevance in humans (19). These findings are mechanistic and ex vivo rather than evidence of comparative clinical efficacy.

The donor-cell stimulator affects both manufacture and product composition. CD40-activated B cells provide a renewable source of APCs with high class II HLA expression and support repeated stimulation during an individualized manufacturing run. Donor PBMCs are easier to obtain initially but vary in cellular composition, viability, and activation state. Neither approach necessarily produces equal responses to every donor mismatch. Release testing should therefore demonstrate donor-directed suppression, exclude generalized hyporesponsiveness, and confirm preservation of third-party responses. The need to collect, qualify, and bank donor leukocytes makes this strategy more practical in planned living-donor transplantation than after unpredictable deceased donation.

### 4.2 Costimulation-blockade-derived products

A related strategy exposes recipient cells to donor antigen while blocking CD28-mediated costimulation. Conventional alloreactive cells are restrained under these conditions and regulatory populations are relatively enriched. Ex vivo costimulation blockade has generated donor-reactive Tregs from patients with advanced kidney disease awaiting transplantation (20). The initial selection phase can be shorter than repeated donor B-cell stimulation, although cellular identity remains protocol dependent and should not be assumed to represent a highly purified conventional Treg product.

The most informative kidney evidence is a three-patient Phase 1/2 pilot in which purified donor-antigen-reactive Tregs were infused 7–11 days after living-donor transplantation. Recipients received tacrolimus, mycophenolate, and corticosteroids without induction. No rejection was found on protocol biopsies, mycophenolate was discontinued at 11–13 months, and all three recipients remained on tacrolimus monotherapy with excellent graft function more than 6 years later. No serious adverse event was attributed to the product (21). These observations support feasibility, safety, and possible immunomodulatory activity, but three patients cannot establish efficacy. In living-donor liver transplantation, a related unpurified costimulation-blockade product was infused in 10 recipients. Seven completed immunosuppression withdrawal, whereas three recipients with autoimmune liver disease developed mild rejection during weaning and resumed therapy (22). This is human non-kidney evidence and should not be extrapolated directly to kidney transplantation.

### 4.3 Peptide-pulsed and indirect-pathway approaches

Indirect-pathway Tregs can be expanded by stimulating recipient cells with autologous APCs loaded with donor-derived peptides. This avoids the need to maintain viable donor cells and presents antigen in the context of the recipient’s HLA molecules. Peptide-loaded monocytes and dendritic cells have been used, sometimes with blockade of CD28 and CD154 signaling (18, 23). The approach may be useful after deceased-donor transplantation when donor leukocytes were not banked, but it replaces a donor-cell constraint with an antigen-selection problem.

Translation depends on more than identifying a mismatched sequence. A candidate peptide must be generated by natural processing, displayed by the relevant recipient HLA molecule at sufficient abundance, and recognized by regulatory precursors that can be expanded. Immunodominance and HLA restriction vary between recipients. The indirect repertoire may evolve with inflammation, antigen availability, and epitope spreading after transplantation. Strong suppression against peptide-loaded APCs *in vitro*, particularly at high peptide concentrations, does not establish that the same peptide is presented at a sufficient density *in vivo*. A narrowly targeted product may also fail to cover direct and semi-direct responses or the breadth of memory responses in a sensitized recipient.

Multiplexing donor-derived peptides could increase coverage, but every added peptide must be naturally processed, presented by an appropriate recipient HLA molecule, and supported by a regulatory precursor population. Additional targets also increase the complexity of product characterization and potency testing. Conversely, limited antigen recognition may still initiate broader control if the activated cells mediate linked suppression against other donor antigens encountered on the same APC or within the graft. This is an important biological rationale, but the magnitude and durability of linked suppression in human transplantation remain uncertain. No direct clinical comparison has established that peptide-based approaches are superior or inferior to donor-cell-based strategies.

#### 4.3.1 ASTRLs as one peptide-based example

Antigen-specific T-cell-enriched regulatory cell lines (ASTRLs) were developed as a post-transplant, donor-cell-independent strategy using peptides from the hypervariable regions of mismatched donor HLA-DR beta chains. Earlier human studies linked responses to donor HLA-DR allopeptides with rejection and chronic dysfunction, while self-restricted donor class II allopeptide-specific regulatory responses were associated with more favorable kidney outcomes (44–46). This work provided a rationale for using a donor class II peptide as an activation anchor. It did not show that one peptide represents the complete donor repertoire.

ASTRL manufacture begins with recipient PBMCs rather than a purified Treg fraction. Cells are repeatedly stimulated for approximately 30–35 days with irradiated autologous APCs loaded with the relevant donor HLA-DR peptide and supplemented with IL-2. In ex vivo studies, ASTRLs suppressed responses to the index peptide and donor cells and exhibited linked suppression to additional donor HLA-DR antigens through the CD39/CD73 adenosinergic pathway (47). Single-cell and multiomic analysis showed antigen-driven CD4^+^ clonal expansion and regulatory-associated programs, while also showing variable CD8^+^ and NK-cell composition (48). The practical advantages are post-transplant manufacture and independence from viable donor cells. The major uncertainties are product heterogeneity, the limited peptide panel studied, between-patient consistency, the contribution of non-CD4 compartments, *in vivo* persistence, dose, and clinical efficacy. ASTRLs have not been infused clinically and remain an ex vivo, preclinical platform.

### 4.4 CAR- and TCR-engineered Tregs

Tregs expressing a transferred T-cell receptor that recognizes a donor-derived peptide presented by recipient MHC class II have promoted long-term graft acceptance in experimental models (24). This approach retains physiological TCR sensitivity and can target the indirect pathway, but eligibility is constrained by both the donor peptide and recipient HLA. Endogenous and transgenic receptor chains can mispair, and unexpected specificity must be assessed. TCR-Treg evidence in transplantation remains preclinical *in vivo*.

Chimeric antigen receptor (CAR)-Tregs recognize an intact surface antigen independently of conventional TCR-MHC engagement. HLA-directed CARs can create a high-frequency donor-reactive population and promote antigen-dependent activation, graft localization, and suppression. HLA-A2 CAR-Tregs have shown greater activity than polyclonal controls in preclinical vascularized allograft models, particularly with adjunctive immunomodulation (25, 26). Eligibility is limited to donor-recipient pairs carrying the selected mismatch, and one CAR cannot represent the full donor antigenic repertoire.

The Phase 1/2 STEADFAST study evaluated autologous HLA-A*02 CAR-Tregs (TX200) in HLA-A*02-negative recipients of HLA-A*02-positive living-donor kidneys (27). The study completed in October 2025, and long-term follow-up continues. Eight recipients received TX200 at approximately 12 weeks after transplantation in the reported dose-escalation cohort. Preliminary conference data described no TX200-related treatment-emergent adverse events, no biopsy-confirmed acute rejection, detection of engineered cells in blood for up to 8 months and in kidney biopsies at 6 months, and reduction to tacrolimus monotherapy in four of five evaluable participants at the higher dose levels (49, 50). These non-peer-reviewed observations support early safety and proof of mechanism. They do not establish operational tolerance or comparative efficacy.

QEL-001 provides complementary human CAR-Treg experience in liver transplantation. Interim sponsor-reported data in March 2026 included nine treated recipients. No drug-related adverse events, dose-limiting toxicities, or serious adverse events were reported. Engineered cells remained detectable for more than 1 year and were enriched in liver tissue. All five recipients who began weaning discontinued tacrolimus and remained off calcineurin inhibition for at least 6 months. Three developed mild to moderate rejection when everolimus was withdrawn or reduced below a trough of 3 ng/mL, and all episodes resolved after immunosuppression was restored (51). The sponsor subsequently prioritized an autoimmune program and sought partnership for QEL-001. This was a strategic development decision rather than a reported safety termination. The liver setting and preliminary, non-peer-reviewed source limit extrapolation to kidney transplantation. **Table 2** summarizes the principal human studies.

**Table 2:** Representative human studies relevant to clinical translation of antigen-specific regulatory cell therapy.

Study, setting, and product	Participants and dose	Timing and background therapy	Follow-up	Safety	Efficacy-related observations and interpretation
ONE Study pooled Phase 1/2A comparison (5) Living-donor kidney Six regulatory cell products, including Treg, dendritic-cell, and macrophage products	38 cell-therapy recipients and 66 reference recipients. Dose and product varied by protocol.	Product-specific peri-transplant administration. Tacrolimus, mycophenolate, and steroids. Cell-therapy trials omitted basiliximab and permitted mycophenolate weaning.	60 weeks	No pooled safety signal compared with the reference group. Fewer infections were reported in the cell-therapy group.	Biopsy-confirmed acute rejection was 16% with cell therapy and 12% in the reference group. Fifteen of 38 cell-therapy recipients were maintained on tacrolimus monotherapy. Heterogeneous products and nonrandomized trials preclude platform-specific efficacy conclusions.
Guinan et al. Phase 1/2 pilot (21) Living-donor kidney Purified donor-antigen-reactive Treg selected with costimulation blockade	n=3. Single individualized infusion. Dose not summarized in the published abstract.	Infused 7–11 days after transplant. No induction. Tacrolimus, mycophenolate, and steroids. Steroids weaned by 14 weeks and mycophenolate at 11–13 months.	>6 years	No serious adverse event attributed to the product.	No rejection on protocol biopsies. All three recipients remained on tacrolimus monotherapy with excellent graft function. Treg-rich lymphoid aggregates were seen at 8 months. The very small uncontrolled cohort supports feasibility and possible activity, not efficacy.
Todo et al. Phase 1/2A pilot (22) Living-donor liver Unpurified donor-cell/costimulation-blockade regulatory product	n=10. Mean total cell dose 23.3 x 10^6^/kg. Mean CD4^+^CD25^+^FOXP3^+^ dose 3.39 x 10^6^/kg.	Infused on postoperative day 13 after cyclophosphamide. Steroids and mycophenolate stopped within 1 month. Calcineurin-inhibitor weaning began at 6 months and was scheduled to finish by 18 months.	Drug-free follow-up 16–33 months in successful recipients at publication.	No significant infusion-related adverse event reported.	Seven recipients completed withdrawal. Three with autoimmune liver disease developed mild rejection during weaning and resumed low-dose therapy. The liver’s greater tolerogenicity limits kidney extrapolation.
Chen et al. mechanistic clinical report (28) Allogeneic HSCT T-allo10 Tr1-enriched product	Clinical tracking in n=3 treated patients. Trial used an individualized donor-derived product.	Administered in an HLA-mismatched HSCT setting. Background conditioning and HSCT care were protocol specific.	Up to 1 year	The report focused on mechanism and persistence rather than a comparative safety analysis.	Transferred Tr1 clonotypes remained detectable for up to 1 year. This is evidence of persistence in a different disease setting and does not demonstrate solid-organ efficacy.
STEADFAST/TX200-TR101 Phase 1/2 (27, 49, 50) Living-donor kidney Autologous anti-HLA-A2 CAR-Treg	13 kidney recipients enrolled. Eight received TX200 in the reported dose-escalation cohort and three controls reached week 12. Four ascending dose levels. Absolute doses were not publicly reported.	Single infusion at approximately 12 weeks. Tacrolimus and mycophenolate with protocol-guided mycophenolate reduction.	Median 88 weeks in the 2025 interim report. Study completed October 2025. Long-term follow-up is active.	No TX200-related treatment-emergent adverse event in the interim report. Registry primary safety results were posted in July 2026.	No biopsy-confirmed acute rejection was reported in the interim cohort. Cells were detected in blood to 8 months and in biopsies to 6 months. Four of five evaluable higher-dose recipients reached tacrolimus monotherapy. Findings are preliminary and non-peer reviewed.
LIBERATE/QEL-001 Phase 1/2 interim report (51) Liver transplant Autologous phenotype-locked anti-HLA-A2 CAR-Treg	n=9 treated. Single infusion. Absolute dose not publicly reported.	Expansion cohort received low-dose ATG before infusion. Baseline tacrolimus plus everolimus, followed by staged weaning.	Engineered cells detected for >1 year. Clinical program includes long-term follow-up.	No drug-related adverse event, dose-limiting toxicity, or serious adverse event reported in the sponsor’s interim disclosure.	All five recipients entering weaning discontinued tacrolimus and remained off it for at least 6 months. Three developed mild to moderate rejection when everolimus was stopped or reduced below 3 ng/mL at 8–9 months. All resolved after immunosuppression was restored. Results are preliminary, non-peer reviewed, and from liver transplantation.

The table is intentionally selective and emphasizes studies that define platform maturity or provide quantitative transplant evidence. Registry and sponsor-reported findings are labeled as non-peer reviewed. DSA outcomes were not consistently reported in the source studies and absence is not inferred when no result is shown. ATG, antithymocyte globulin; CAR, chimeric antigen receptor; DSA, donor-specific antibody; HSCT, hematopoietic stem-cell transplantation; Treg, regulatory T cell.

### 4.5 Tr1 and other antigen-specific regulatory products

Not all antigen-specific regulatory products are conventional FOXP3^+^ Tregs. IL-10-conditioned tolerogenic APCs can generate Tr1-enriched products that suppress through IL-10, CTLA-4, PD-1, and related pathways. In allogeneic hematopoietic stem-cell transplantation, T-allo10 products showed alloantigen-driven clonal restriction and persistence of Tr1 clonotypes for up to 1 year in three treated patients (28). This is human evidence in a different clinical setting and regulatory lineage. Comparative characterization also shows that products collectively described as alloantigen-specific differ substantially in phenotype, repertoire, potency, and manufacturability (29).

## 5 Culture conditions and manufacturing trade-offs

Antigen determines which cells are selected, whereas the starting population and culture environment shape what those cells become. Most conventional products begin with CD4^+^CD25^high^CD127^low^ cells isolated from peripheral blood or leukapheresis. Naive CD45RA^+^ Tregs may offer greater proliferative capacity and lineage stability, while memory Tregs may contain more pre-existing donor reactivity. Cord blood and pediatric thymus provide alternative sources with high proliferative potential but are not generally autologous options for adult kidney recipients (30–33). Greater sorting purity reduces contaminating conventional cells but also lowers starting yield, increases cost, and may narrow the repertoire.

The concentration and timing of IL-2 supplementation during culture are important determinants of expansion and product composition. Insufficient IL-2 limits yield. In an unsorted culture, however, IL-2 can support activated conventional T cells, CD8^+^ cells, and NK cells. Rapamycin preferentially restrains conventional T-cell outgrowth and is widely used to enrich FOXP3^+^ cells, although it may slow expansion. TGF-beta and retinoic acid can promote FOXP3 induction or stability, and IL-15 has been incorporated into selected alloantigen-specific protocols (34–36). These additives are not interchangeable. A condition that improves purity in a conventional Treg product may alter a deliberately heterogeneous product.

Many platforms therefore use a two-stage process, with antigen-driven selection followed by nonspecific expansion such as CD3/CD28 stimulation. This can generate a clinical dose but may dilute donor-reactive clonotypes, favor faster-growing subsets, or destabilize phenotype. A recent protocol combining alloantigen selection with IL-2, IL-15, retinoic acid, rapamycin, TGF-beta, and subsequent polyclonal expansion produced a highly enriched CD25^+^CD27^+^CD70^-^ population with substantial FOXP3-TSDR demethylation and retained function during inflammatory challenge (37). The result illustrates a manufacturing possibility rather than a cross-platform efficacy comparison.

The unresolved trade-offs differ by platform (**Table 1**). Donor-cell products exchange broad antigenic coverage for individualized donor-cell procurement and longer manufacture. Peptide products simplify donor access but add HLA restriction and antigen-validation requirements. Engineered products provide defined receptor specificity and avoid prolonged antigen-selection cultures, but require gene transfer, vector testing, construct-specific potency assays, and long-term follow-up. Across platforms, larger expansion can alter clonotype representation and product composition. It may therefore affect more than final dose. Few head-to-head studies and no universal potency assay permit a valid ranking of manufacturing systems (29, 38–40).

Translation requires a closed, reproducible process, robust cryopreservation, and a potency assay linked to the proposed mechanism. Gas-permeable bioreactors can support economical scale-up (41), but expansion is not itself a release criterion. A credible release strategy should address identity, viability, sterility, endotoxin and mycoplasma contamination, and product-specific function. Additional measures may include FOXP3 and TSDR stability where relevant, donor-specific suppression, preservation of third-party responses, cytokine and cytotoxicity profiles, antigen-driven clonality, post-thaw recovery, and the contribution and consistency of each compartment in a heterogeneous product.

## 6 Interaction with conventional immunosuppression

An infused regulatory product cannot be evaluated independently of its immunosuppressive environment. Calcineurin inhibitors reduce NFAT-dependent IL-2 production and may limit survival or expansion of conventional Tregs. Cross-sectional transplant data associate higher calcineurin-inhibitor exposure with lower Treg number and weaker phenotype or function, while rapamycin-based treatment is associated with more favorable Treg measures (52). Conversion from tacrolimus to sirolimus in kidney recipients also shifted immune profiles toward a higher regulatory-to-effector balance (53). These studies concern endogenous Tregs and do not prove that mTOR inhibition is superior for every infused product.

Induction and corticosteroid effects are similarly context dependent. Rabbit antithymocyte globulin can produce relative enrichment or expansion of circulating Tregs during immune reconstitution, whereas CD25 blockade can transiently reduce phenotypically defined CD25^+^ Tregs (54). Lymphocyte depletion may create space and cytokine availability for an infused product, but it can also alter trafficking, homeostatic proliferation, infection risk, and the composition of recovering effector cells. Corticosteroid dose and timing may affect both endogenous and infused populations.

Low-dose IL-2 can preferentially expand Tregs in chronic graft-versus-host disease (55), but this evidence is outside solid-organ transplantation. In kidney recipients, IL-2 could also influence activated conventional T cells and NK cells, particularly in the inflammatory early post-transplant period. Its use as an adjunct therefore remains investigational. The accompanying regimen should be selected for the product, timing, and trial objective. Current data do not justify one universal immunosuppressive regimen for conventional Tregs, Tr1-enriched products, heterogeneous regulatory lines, and engineered-receptor Tregs.

## 7 Safety considerations

Risks shared by ex vivo-expanded regulatory products include infusion reactions, expansion or persistence of contaminating conventional effector cells, loss of regulatory phenotype, and unintended or excessive systemic suppression. Infection, viral reactivation, impaired tumor surveillance, and malignancy therefore require prospective surveillance even when the intended activity is donor focused. Product purity, lineage or functional stability, cytokine and cytotoxicity profiles, and preservation of third-party responses are relevant release and follow-up measures.

Engineered products add construct-specific risks. These include unexpected antigen recognition, immunogenicity of receptor components, transgene or vector-related events, loss or silencing of the engineered program, and long-term genomic safety. Tonic CAR signaling can drive human Treg exhaustion, and the choice of costimulatory domain changes CAR-Treg function (56, 57). TCR-engineered products additionally require control of endogenous-transgenic TCR mispairing. Long-term surveillance should therefore combine clinical outcomes with receptor persistence, phenotype, clonal behavior, and vector-specific assessments. Endogenous TCR expression in an autologous CAR-Treg product is not, by itself, a reason to describe graft-versus-host disease as a principal expected toxicity.

## 8 Timing, persistence, and durability

Pretransplant administration has a theoretical advantage because it can intervene before initial alloimmune priming and, with a living donor, provides a predictable manufacturing interval. It also permits donor-cell collection before surgery. Its limitations include restricted applicability to planned transplantation, use of starting cells obtained in the uremic state, and absence of the graft as a continuing source of antigen. Early post-transplant administration places cells in an antigen-rich environment, but ischemia-reperfusion injury, lymphocyte depletion, corticosteroids, calcineurin inhibition, and limited IL-2 may compromise survival or function.

Delayed administration avoids the most inflammatory perioperative period and is feasible after deceased donation. It may be particularly suitable for stable recipients in whom the objective is immunosuppression reduction. The trade-off is that effector-memory populations, donor-specific antibody, or chronic structural injury may already be established. Pretransplant, early post-transplant, and delayed approaches are therefore complementary hypotheses rather than a proven ranking of timing strategies. Repeat or staged dosing may warrant study for products that can be remanufactured, but current clinical evidence does not establish that it is required.

Durability should be defined through common domains with platform-specific measurements. Cellular persistence can be measured by an engineered receptor, vector sequence, genetic marker, or identifiable clonotype. Regulatory stability can include FOXP3 expression and TSDR demethylation for conventional Tregs, but these measures are not appropriate for Tr1 products and may not capture the behavior of every engineered platform. Antigen-responsive function can be assessed with donor-specific recall or suppression assays together with preservation of third-party responses. Tissue engagement can include graft localization, protocol or for-cause histology, molecular injury signatures, and donor-specific antibody. Clinical durability requires stable graft function and sustained immunosuppression reduction without rejection, infection, malignancy, progressive fibrosis, or loss of protective immunity.

An illustrative trial schedule would assess acute safety and distribution through day 28, cellular persistence and product identity at approximately 1, 3, and 6 months, and donor-specific function, biopsy or molecular injury, donor-specific antibody, graft function, infectious events, and immunosuppressive exposure at 6 and 12 months and then through at least 24 months. The precise schedule should follow the expected biology of the product. High-dimensional clonotype and single-cell assays are valuable mechanistic endpoints but may remain exploratory in larger trials. Intragraft Treg-rich structures and IL-35-associated exosome-mediated regulation illustrate possible tissue-level mechanisms, but they do not establish that an infused product created a durable network (42, 43). **Figure 1** summarizes the treatment contexts and measurement domains.

**Figure 1:**
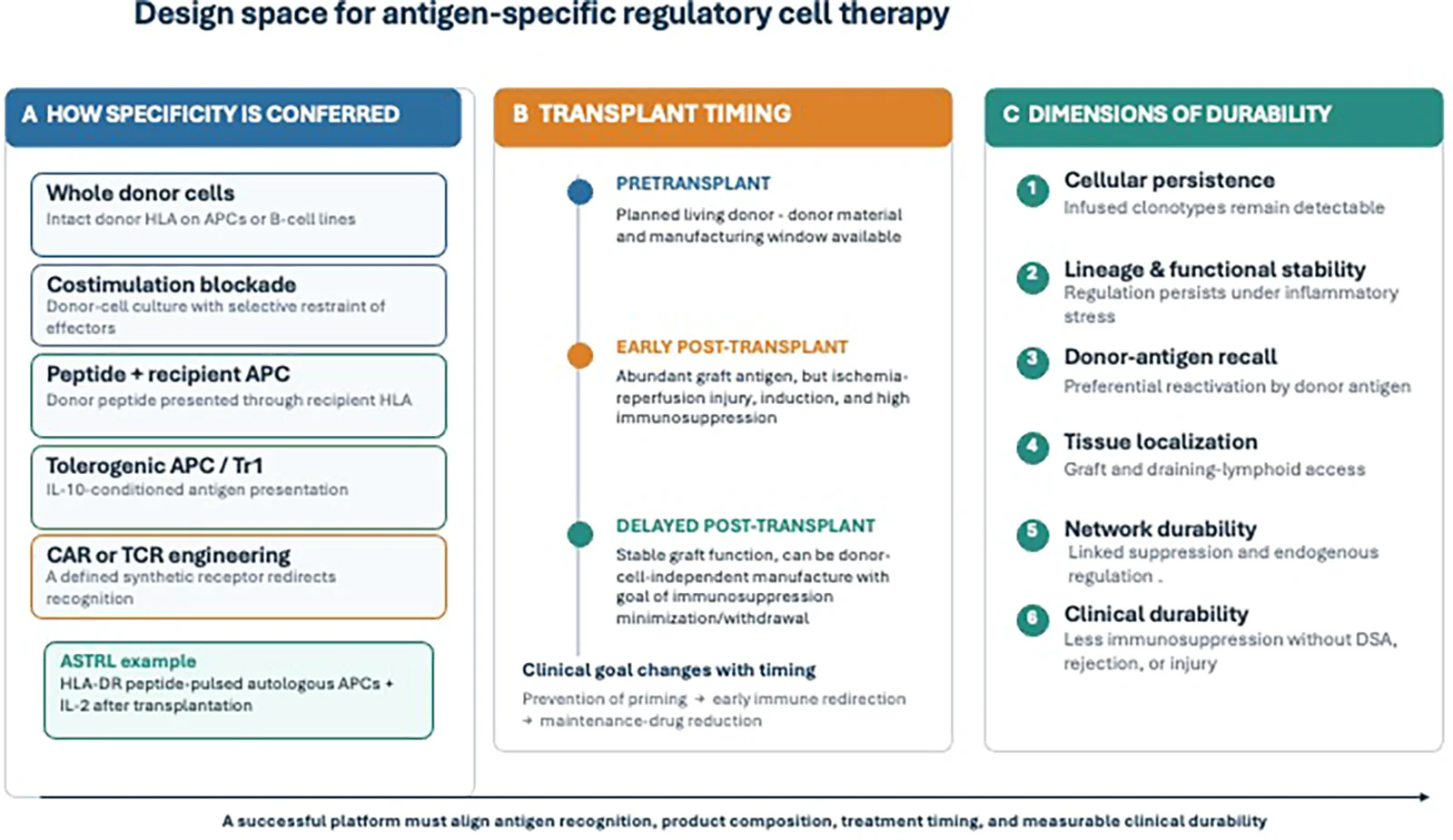
Design space for antigen-specific regulatory cell therapy.

## 9 Discussion

No single platform can yet be designated as best for kidney transplantation. A clinically useful framework begins with the intended setting. Planned living-donor transplantation provides time to obtain donor material and manufacture donor-cell-expanded or costimulation-blockade products. It also permits eligibility testing for a receptor-defined product before surgery. Deceased-donor transplantation favors products that can be banked, manufactured without viable donor cells after HLA typing, or delivered later. These logistical advantages do not establish biological superiority.

Stable late recipients being considered for immunosuppression reduction differ fundamentally from highly sensitized recipients or patients with established donor-specific antibody and chronic alloimmune injury. In a stable recipient, a delayed donor-independent product can be tested against measurable minimization and surveillance endpoints. In a sensitized recipient, broad memory and antibody responses may make narrow antigen coverage insufficient, but no platform has been compared adequately in this setting. For established DSA or chronic injury, there is no evidence that an antigen-specific regulatory product reverses structural damage or active antibody-mediated injury. Such trials should be framed as experimental adjunctive strategies and should include histologic and molecular injury endpoints rather than assume tolerance induction.

The term antigen-specific Treg currently encompasses products that differ in antigen source, recognition pathway, cellular composition, and mechanism. The proposed nomenclature and evidence labels make those differences explicit. Antigen-specific products may act through direct suppression, linked suppression, metabolic control, altered APC function, and recruitment of endogenous regulation. The relative contribution and durability of these mechanisms remain incompletely defined. Cross-platform potency assays, transparent reporting of manufacturing attrition, and prospective measurement of cell fate and graft injury are needed before platform selection can become prescriptive.

Future trials should match the product to a defined clinical objective rather than use a uniform tolerance endpoint. Prevention of early priming, rescue of active alloimmunity, and reinforcement of regulation in a stable graft are different objectives and may require different cells, timing, adjunctive therapy, and dosing. The near-term clinical goal should be safe and durable immunosuppression minimization. Operational tolerance may be achievable in selected recipients, but it must be demonstrated by sustained graft stability without immunosuppression rather than inferred from cellular persistence or a favorable biopsy.
